# Hydrodynamic assisted multiparametric particle spectrometry

**DOI:** 10.1038/s41598-021-82708-0

**Published:** 2021-02-11

**Authors:** Alberto Martín-Pérez, Daniel Ramos, Marina L. Yubero, Sergio García-López, Priscila M. Kosaka, Javier Tamayo, Montserrat Calleja

**Affiliations:** Bionanomechanics Lab, Instituto de Micro y Nanotecnología, IMN-CNM (CSIC), Isaac Newton 8 (PTM), E-28760 Tres Cantos, Madrid, Spain

**Keywords:** Nanoscale biophysics, Biosensors, Microfluidics, Imaging and sensing, Mechanical engineering, NEMS, Sensors

## Abstract

The real-time analysis of single analytes in flow is becoming increasingly relevant in cell biology. In this work, we theoretically predict and experimentally demonstrate hydrodynamic focusing with hollow nanomechanical resonators by using an interferometric system which allows the optical probing of flowing particles and tracking of the fundamental mechanical mode of the resonator. We have characterized the hydrodynamic forces acting on the particles, which will determine their velocity depending on their diameter. By using the parameters simultaneously acquired: frequency shift, velocity and reflectivity, we can unambiguously classify flowing particles in real-time, allowing the measurement of the mass density: 1.35 ± 0.07 g·mL^-1^ for PMMA and 1.7 ± 0.2 g·mL^-1^ for silica particles, which perfectly agrees with the nominal values. Once we have tested our technique, MCF-7 human breast adenocarcinoma cells are characterized (1.11 ± 0.08 g·mL^-1^) with high throughput (300 cells/minute) observing a dependency with their size, opening the door for individual cell cycle studies.

## Introduction

High throughput sorting and characterization of individual micro and nanoparticles in physiological environments presents a matter of essential interest in many different subjects such as environmental control, clinical assays, nanotoxicology or cell biology. For these purposes, the use of microfluidic devices has been demonstrated as a robust technique for label-free passive particle sorting, performing with a throughput of up to 10^7^ particles/min, just by taking advantage of the forces exerted by the moving fluid on the particle^1^. These hydrodynamic forces depend on particle shape and size, which eventually results in particles moving along with the fluid^2^. Despite allowing particle analysis based on geometrical properties, static microfluidic devices cannot perform particle discerning in cases of same-shaped particles composed of different materials. Therefore, it needs to be combined with other techniques (e.g. optical methods measuring refractive index) to discern among same-shaped particles^3–7^. On the other hand, nanomechanical resonators have been demonstrated as a powerful analysis tool given they can measure multiple physical properties (force^8^, mass^9^, stiffness^10–12^, optical absorption^13^, etc.) with extremely high sensitivity, by tracking the changes in the mechanical resonance frequency of a vibrating structure. Nevertheless, this high sensitivity is ruined by the viscous dragging forces when operated immersed in liquid due to the displaced mass during the oscillation cycle^14^. Suspended microchannel resonators (SMR)^15^ were developed to analyze particles in liquid environments overcoming the problems outlined before by merging microfluidics and nanomechanics. SMR approach consists of a nanomechanical resonator with an inner integrated microchannel, allowing the resonator to vibrate in a gaseous or vacuum environment, while analytes are characterized in the flowing liquid^16^. Since the viscous dragging forces vanish as the liquid now is placed inside the resonator, there is an enhancement of the buoyant mass resolution: up to 10 attograms in the current state of the art devices^17^ operated in vacuum.

Among the SMR devices, transparent microcapillary resonators (TMR) not only combine a nanomechanical resonator with a microfluidic channel but also allow the optical probing of the flowing particles. These devices have been demonstrated in previous works as an interesting alternative to cantilever-type SMRs for mass sensing^18–23^, introducing a new source of information about the particle based on its optical characterization^24,25^. This double mechano-optical particle detection technique has been proved in recent work as a highly reliable technique for particle discerning, even in cases of particles of similar masses. Despite this high reliability, it was still necessary to have prior information about particle size acquired by other techniques to calculate some intensive parameters of the particles, e.g. density or refractive index. In particular, density has been demonstrated as an interesting parameter for cell life-cycle characterization in previous works using SMR^26,27^ due to the relation of cell density with biological activity^28–31^. Currently existing techniques require measuring the buoyant mass of the same cell in two different-density media to measure cell density, dramatically reducing the measurement throughput. In this work, we propose the use of hydrodynamic particle focusing and multiparameter sensing to obtain information about the particle velocity (and, hence, about its size) by analyzing the mechanical frequency shift. The frequency fluctuation reaches a value of about 10^–7^ for integration times of about 150 ms, which implies a mass resolution of 0.6 pg and size resolution of about 100 nm. This velocity measurement is simultaneously acquired to buoyant mass and reflectivity, allowing a high-throughput triple-parameter particle characterization technique. By combining these three parameters, we can measure either size or mass density, obtaining a result of 1.35 ± 0.07 g·mL^-1^ for PMMA particles and 1.7 ± 0.2 g·mL^-1^ for silica particles, which perfectly agrees with their nominal values. Once we have tested our technique with calibration particles, we have measured the size and density of individual MCF-7 human breast adenocarcinoma cells (1.11 ± 0.08 g·mL^-1^), observing a dependency with their size. These results open the door for individual cell cycle studies.

## Results

### Device characterization

**Figure 1:**
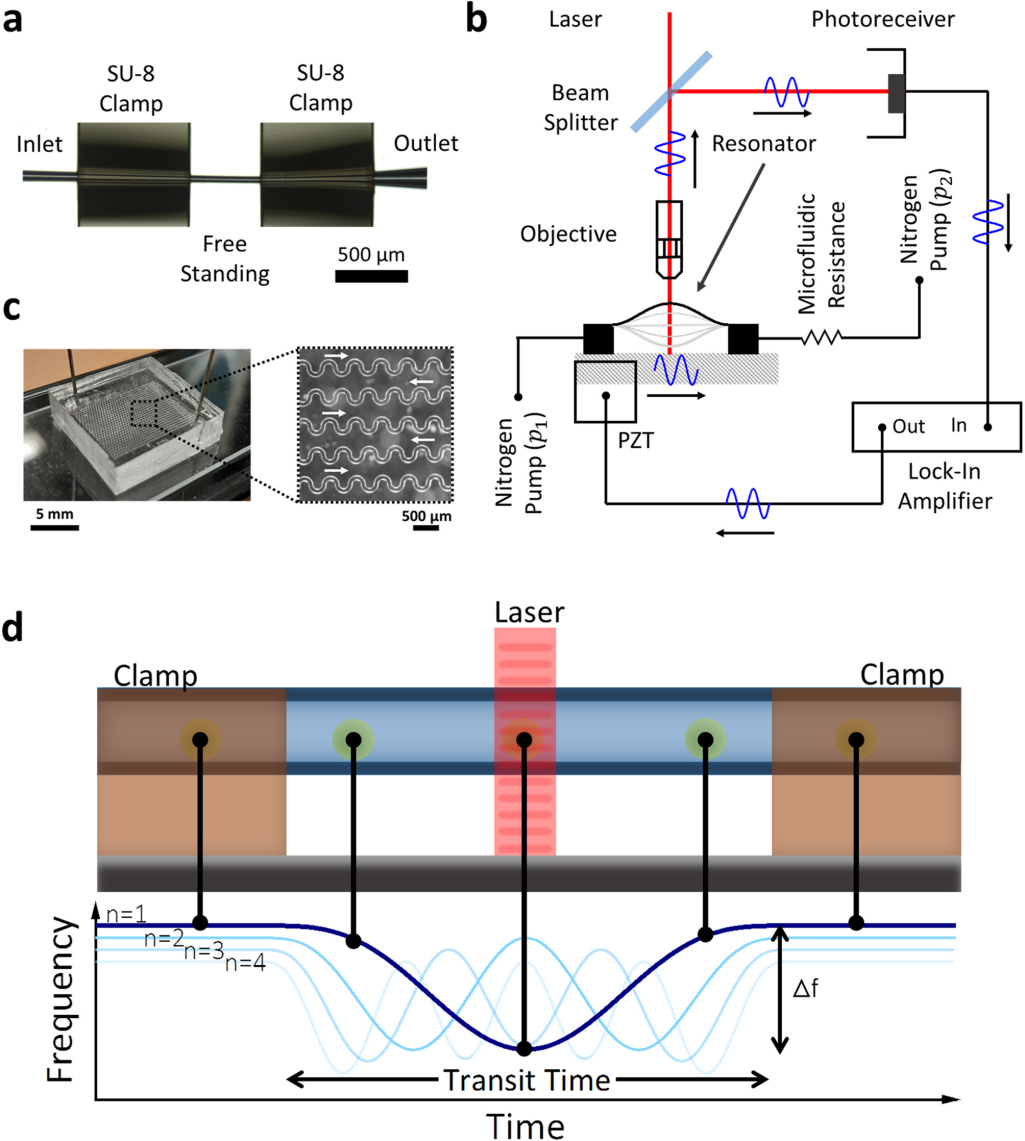
Device characterization. (**a**) Optical micrograph showing a transparent microcapillary resonator. (**b**) Schematics of the experimental setup. The homemade optical interferometric system and details of the microfluidic setup. (**c**) Image of the PDMS microfluidic resistance. White arrows indicate flow direction. (**d**) Working principle of the nanomechanical sensing using transparent microcapillary resonators, showing the frequency shifts that the particle produces for the first four mechanical modes.

We use a TMR of 44 µm outer diameter and 34 µm inner diameter obtained by locally elongating a 350 µm outer diameter fused silica capillary with a wall thickness of 50 µm. These pulled microcapillaries are integrated on a silicon substrate and doubly clamped with photolithographed polymeric (SU-8) pads, obtaining a free-standing region of 500 µm length, Fig. 1a, in which the hollow microchannel can mechanically oscillate in a guitar-string mode. The fabrication of the device is based on a thermal stretching technique described elsewhere^24^. The mechanical modes of the capillary are excited by means of a piezoelectric actuator, while its resonance frequency is tracked in real-time by means of a home-made interferometric readout system and a lock-in amplifier (Fig. 1b). This interferometric system also allows tracking the reflected light power by the fused silica resonator to obtain information about the optical properties of the flowing analytes by analyzing in real-time the scattered light^24,25^. Moreover, the device is pressurized by means of a nitrogen pressure pump (Fluigent INC, MFCS-EZ-07000001), which allows flow control by setting a pressure difference. In order to increase the control of the flow rate, the outlet of the device is followed by a home-made microfluidic resistance, a rectangular cross section polydimethylsiloxane (PDMS) channel of 40 µm × 75 µm × 72 cm performing a microfluidic resistance of 7.8 mbar·s·nL^-1^, Fig. 1c. Finally, the outlet end is connected to the pump allowing to set minimum pressure difference of 5 mbar between the inlet of the TMR device and the outlet of the PDMS resistance.

### Nanomechanical response

A particle flowing through the free-standing region of the device shifts the resonance frequency signal (Fig. 1d). The amplitude of this dip (Δf) depends on particle buoyant mass while its width (transit time, t_T_) depends on particle velocity. By assuming a constant velocity, the flowing particles, whose diameter is very small compared to the length of the suspended region, probes the mechanical mode; therefore, frequency shift signal tracked in the lock-in amplifier as a function of time for the n-th mechanical mode can be written as

1 $$ \begin{aligned}{f}_{n}\left(t\right)&={f}_{n0}+\frac{\Delta f}{{\psi }_{n,max}}\left[cosh\left[\frac{(2t-2{t}_{0}+{t}_{T}) {\beta }_{n}}{2{t}_{T}}\right]-cos\left[\frac{(2t-2{t}_{0}+{t}_{T}) {\beta }_{n}}{2{t}_{T}}\right]\right.\\&\quad\left.+\frac{cos{\beta }_{n}-cosh{\beta }_{n}}{sin{\beta }_{n}-sinh{\beta }_{n}}\left[sin\left[\frac{(2t-2{t}_{0}+{t}_{T}) {\beta }_{n}}{2{t}_{T}}\right]-sinh\left[\frac{(2t-2{t}_{0}+{t}_{T}) {\beta }_{n}}{2{t}_{T}}\right]\right]\right]\end{aligned} $$

Being f_n_(t), the frequency of the n-th flexural mode as a function of time, f_n0_ the natural resonance frequency of the mechanical mode of the resonator, ${\psi }_{n,max}$ the value of the normalized mechanical mode shape at its maximum amplitude, t_0_ the time in which the particle is in the middle of the free-standing region and ${\beta }_{n}$ the mode eigenvalue (first four eigenvalues are ${\beta }_{n}=4.7300, 7.8532, 10.9956, 14.1372$). Blue lines in Fig. 1d show the frequency shift induced by a particle on the first four flexural modes of the TMR. Without limiting the generality of the foregoing, we are going to study the fundamental mode. As it was stated before, the lock-in amplifier allows us to track the capillary resonance frequency as a function of time. Therefore, fitting the temporal frequency shifts to Eq. (1) allows obtaining either particle buoyant mass through the Δ*f* fitting parameter and the transit time, t_T_. The particle velocity is then straightforwardly obtained by dividing the length of the suspended region, which is set by the fabrication process, by the transit time obtained from the fitting.

### Analytical model

**Figure 2:**
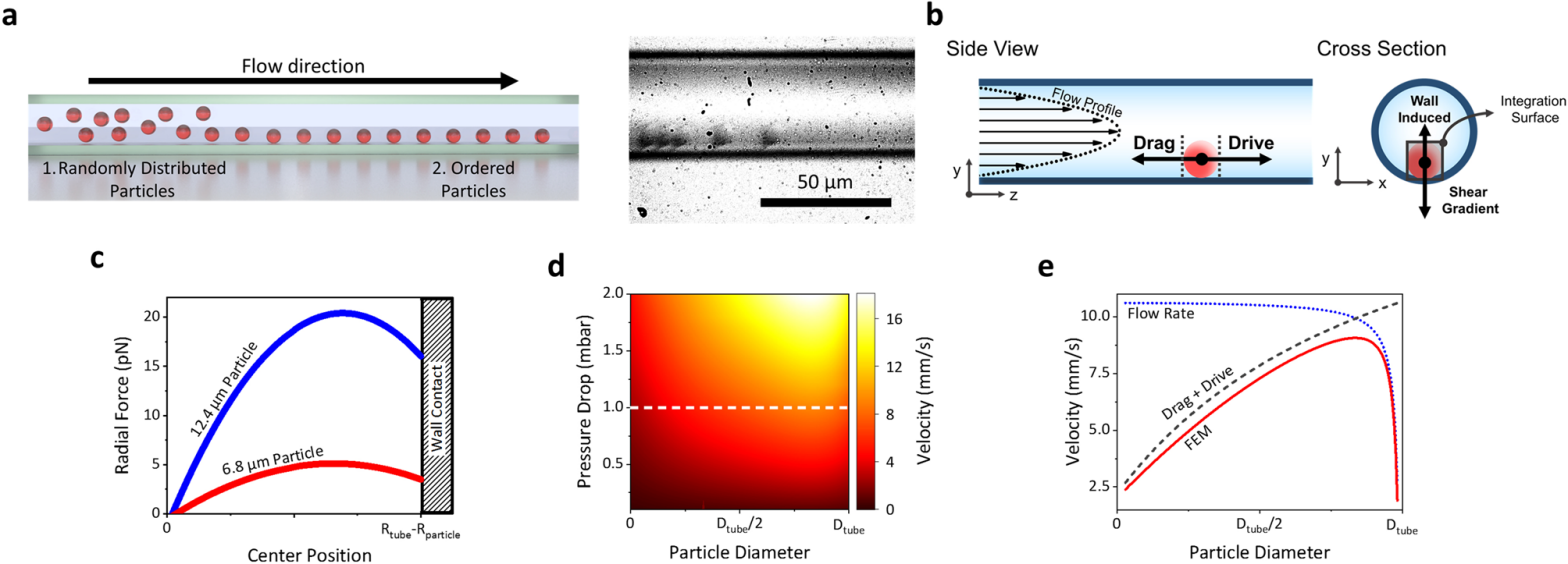
Theoretical framework. (**a**) Schematics of microfluidic inertial particle focusing (left, made by open- source software Blender, https://www.blender.org/) and optical micrograph of 6.8 µm silica particles passing through the free-standing region of the device (right). (**b**) Schematics of the calculation area, cross-section and side view. (**c**) FEM Simulations of the force exerted by the fluid in the radial direction of the tube as a function of the radial position (being R_tube_ the radius of the tube and R_particle_ the radius of the particle) for a flow rate of 9 nL/s. (**d**) Terminal velocity as a function of pressure drops and particle diameter obtained by FEM. (**e**) Terminal velocity as a function of particle diameter for a constant pressure drop (white dashed line in d) compared to the effect of flow rate reduction, dotted line, and the effect of dragging and driving force balance, dashed line.

The particle movement is the result of the force exerted by the liquid on the surface of the particle. Therefore, hereinafter we are going to study the dependency of the hydrodynamic force on the particle size, which will allow to classify particles by their radii. In order to experimentally demonstrate this, an aqueous suspension of microparticles is introduced in the TMR device. If particles were just subjected to the dragging forces of the fluid in the flow direction, they should follow the parabolic velocity profile of a laminar flow in a cylindrical tube^32^. However, previous works on microfluidics^1,2^ have demonstrated that, within a laminar flow, each particle also suffers a displacement orthogonal to flow direction, see schematics and optical micrograph in Fig. 2a. This is the consequence of the balance between two lifting forces exerted by the fluid on the moving particle: shear gradient (centrifugal) and wall-induced lift (centripetal), Fig. 2b. In order to calculate the equilibrium position of the particles in our experiment, we perform finite element simulations (FEM, COMSOL Multiphysics) to calculate the net force on the y-direction on 6.8 µm (red curve in Fig. 2c) and 12.4 µm (blue curve in Fig. 2c) diameter spherical particles. We simulate a cylindrical tube whose dimensions mimic the experimental free-standing region and set a pressure difference between its extremes. The simulations reveal that the dominant force is always centrifugal (Fig. 2c), hence, in this configuration the particle equilibrium position is wall contact. This result is in good agreement either with previous works on inertial focusing with high particle diameter/channel width ratio^33^ and with our experimental observations, as seen from optical micrograph in Fig. 2a. Therefore, the randomly distributed particles introduced in the capillary tube will be subjected to lifting forces, making all particles precipitate on the capillary wall. Once precipitated, the drive and drag force on the particle reach a steady state. Assuming a Newtonian fluid (incompressible liquid and uniform viscosity) within the laminar flow regime, the drag force depends on particle velocity following the Stoke’s Law, ${F}_{stokes}=A({R}_{particle}) 6\pi \mu {R}_{particle}v$, where ${R}_{particle}$ is the radius of the particle, $A({R}_{particle})$ a geometrical correction factor and $v$ the particle velocity. Since our device consists of a cylindrical tube, the velocity profile follows a paraboloidal shape, which can be written in cartesian coordinates as^34^

2 $${u}_{z}(x,y)={u}_{0}\left(1-\frac{{x}^{2}+{y}^{2}}{{{R}_{tube}}^{2}}\right)$$

where ${u}_{0}$ is the fluid velocity at the axis of the tube, and ${R}_{tube}$ the inner radius of the tube. According to Navier–Stokes equation, the pressure is then given by $p=-\frac{4\mu {u}_{0}}{{{R}_{tube}}^{2}}z$, where $\mu$ is the dynamic viscosity, and x, y and z the cartesian coordinates, Fig. 2b. The force exerted by the fluid on the particle can be calculated by means of the anisotropy of the integral of the stress tensor, ${\sigma }_{ij}=-p{\delta }_{ij}+\mu \left(\frac{\partial {u}_{i}}{\partial {x}_{j}}+\frac{\partial {u}_{j}}{\partial {x}_{i}}\right)$, over the surface of the sphere ${F}_{z}=\oint-{\sigma }_{zi}dS$. By assuming a flat surface instead of a spherical surface integration and neglecting the perturbation in the flow profile caused by the particle, the z-component of the hydrodynamic force, along the tube long axis, can be written as

3 $${F}_{z}=\frac{4\mu {u}_{0}{{R}_{particle}}^{2}}{{{R}_{tube}}^{2}}\left({R}_{tube}-{R}_{particle}\right)$$

By simply equaling the force given in Eq. (2), with the Stokes’ law, the z-component of the particle velocity is given by

4 $${v}_{z}=\frac{2{u}_{0}}{3\pi A({R}_{particle}) {{R}_{tube}}^{2}}{R}_{particle}({R}_{tube}-{R}_{particle})$$

Thus, the particle velocity parabolically depends on its radius (as bigger particles will be exposed to higher fluid velocities). Moreover, the fluid velocity at the axis can be related to pressure difference, $\Delta p=-\frac{4\mu {u}_{0}}{{{R}_{tube}}^{2}}L$, implying a linear dependency between particle velocity and pressure difference. For a better understanding of the relationship between particle diameter and velocity we have performed time-dependent 2D FEM simulations of an unconstrained particle inside a tube (mimicking the experimental suspended channel). In these simulations, we set a pressure difference between the tube ends (pressure drop) with a particle in the middle of the axial position. In order to avoid convergence problems, the particle is placed 1 µm above the capillary wall. At the beginning of the time-dependent simulations, the particle starts to move increasing its velocity until it reaches a constant value, terminal velocity. This terminal velocity is experimentally measured as particle mean velocity. The simulation was run to obtain the terminal velocity for different particle diameters (ranging from 0.2 µm up to the tube inner diameter) and pressure drops (ranging from 0 up to 2 mbar), Fig. 2d. Constant pressure simulations reveal that for small particle diameter, smaller than ¾ of the tube inner diameter, the terminal velocity continuously increases with particle diameter, then, for bigger diameters, it dramatically drops (Fig. 2e). The terminal velocity dependency on the particle diameter can be understood as a balance between two opposite factors contributing to particle velocity: fluid transferring momentum to the particle (drag and drive forces equilibrium), which increases with the particle cross section, and flow rate decrease caused by the particle partially obstructing the channel, inversely proportional to the difference between tube and particle cross sections, Fig. 2e. Moreover, in constant diameter simulations while varying pressure drops, the terminal velocity linearly depends on pressure drop, indicating that the driving force increases with flow rate as expected.

### Hydrodynamic particle spectrometry

**Figure 3:**
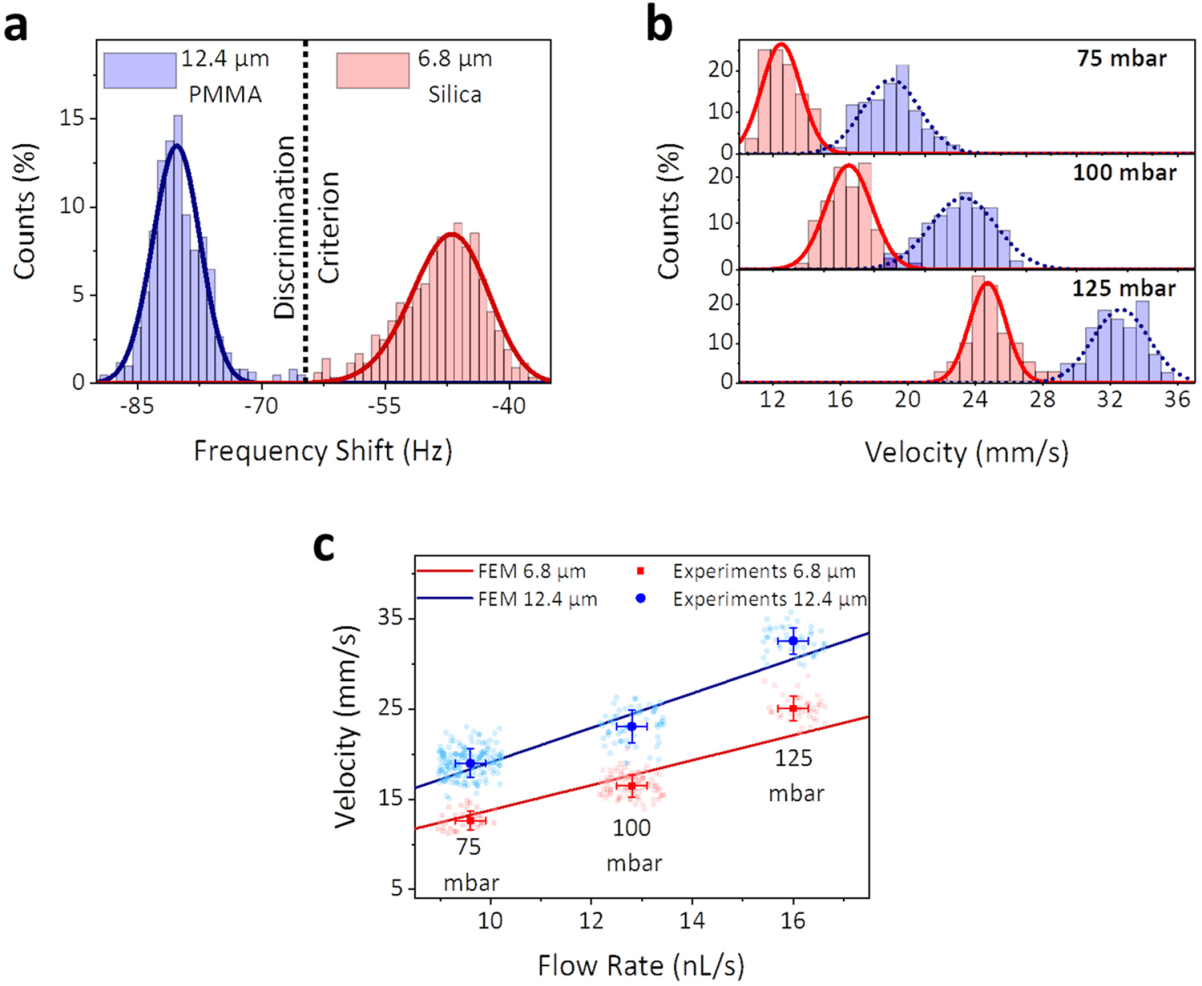
Experiments with particle mixtures**.** (**a**) Frequency spectra for the particle mixture (bars) and fits to gaussian distributions (solid lines). We set -65 Hz as discrimination criterion between two particle populations. (**b**) Experimental histograms of measured velocity for all particles for 3 differential pressures. (**c**) Experimental data (points) and FEM simulations (solid lines) of the particle velocity as a function of the applied flow rate for the PMMA particles (blue) and silica particles (red).

To experimentally demonstrate the theoretical results, we flow through the device a mixture of two different spherical microparticles suspended in an aqueous medium: 6.8 ± 0.4 µm diameter silica particle and 12.4 ± 0.2 µm diameter poly(methyl methacrylate) (PMMA) (Microparticles GmbH, Germany). This experiment is repeated applying different flow rates (i.e., different pressure differences). Both particles differ either in diameter or buoyant masses (PMMA particles are expected to present a buoyant mass 1.7 times higher than silica particles in ultrapure water), hence, we can associate each event to a certain particle type. We track the frequency shifts produced by the passing particles for different pressure drops obtaining the frequency shift and velocity of each individual particle. Given the actual dimensions and mass densities of the materials, we set a threshold to distinguish between both particle populations, every single particle producing a frequency shift lower than -65 Hz is a 12.4 µm PMMA particle, over this value the particles are 6.8 µm silica (Fig. 3a). Regarding the particle velocity, we can discern between two populations, being the slower particles those with a smaller diameter, (silica particles), while the faster particles are those with the larger diameter (PMMA particles), histograms in Fig. 3b. The velocity difference between the two particle populations becomes bigger as the pressure difference is increased. To compare experimental results and simulations we plot particle velocity as a function of flow rate, blue and red symbols in Fig. 3c for PMMA and silica particles respectively. In the case of the simulations (solid lines in Fig. 3c) the flow rate is calculated from the velocity field in the fluid while for the experiments flow rate is estimated by considering the pressure difference set and the resistance of 7.8 mbar·s·nL^-1^ of the capillary device connected to the PDMS resistance. Experiments and simulations are in very good agreement: for a given size, particle velocity increases linearly with the pressure difference while the ratio velocity/pressure depends on particle size.

### Multiparametric particle spectrometry

**Figure 4:**
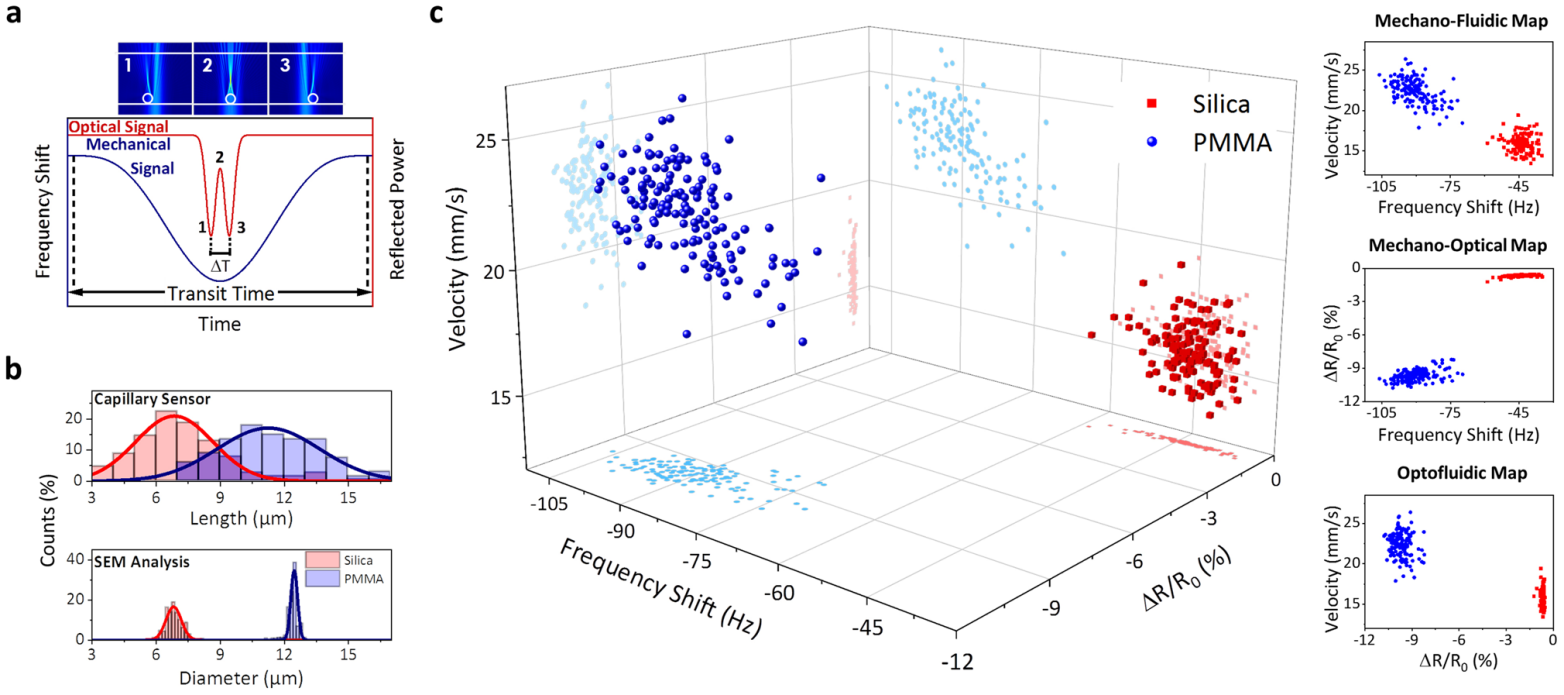
Multiparametric particle spectrometry**.** (**a**) Schematics of the particle length measuring principle. Up. Electric field distribution for the particle passing under the laser spot at different positions (1–3). Down. Mechanical (blue line) and optical (red line) signal expected for this particle. (**b**) Up. Particle size distribution measured for a sample of 67 PMMA particles and 192 Silica particles. Down. Particle size distribution measured by SEM images inspection. (**c**) Left. 3D scatter plot (particle velocity, frequency shift and reflectivity change). Right. 2D projections for the mixture of PMMA and silica particles being flown at 100 mbar.

The TMR and the readout system also allow tracking the reflected light power (optical signal) by the device^25^. Hence, when a particle passes under the illuminated region by the laser spot it produces a dip in this optical signal due to the scattered light, which can be used to obtain additional information about the particle. This dip in the optical signal consists of a double peak whose local maximum corresponds to the particle positioned in the center of the spot while its local minima correspond to the scattered light at the particle border: entering and leaving the illuminated spot area^25^. Hence, given the particle velocity obtained by fitting the mechanical signal, the particle diameter can be calculated by measuring the time-difference between the two local minima in the optical signal (Fig. 4a). We obtain a size distribution of 11 ± 2 µm for PMMA particles and 7 ± 2 µm for silica particles (upper chart in Fig. 4b), these values reproduce the results obtained by analyzing scanning electron microscope (SEM) images of the same populations 12.4 ± 0.2 µm for PMMA particles and 6.8 ± 0.4 µm for silica particles, lower chart in Fig. 4b. The large dispersion attained due to the measurement errors inherent to the proposed methodology are still acceptable, considering that values are measured with very high throughput and in liquid environment (> 300 particles/min), compared to the SEM images that are acquired in high vacuum with very low throughput ($\sim$ 1 image/minute). Eventually, this measuring method allows the simultaneous measurement of three different and independent parameters of every single particle (buoyant mass, particle velocity and reflectivity change, $\Delta R/{R}_{0}$) with extremely high throughput, up to 300 particles per minute. When plotted in a 3D scatter diagram, the previous particle mixture can be unambiguously discerned (Fig. 4c) performing a highly reliable particle discerning based on three independent parameters. The simultaneous measurement of the mass and the size of the particle allows the calculation of the mass density, giving a result of 1.35 ± 0.07 g·mL^-1^ for silica particles and 1.7 ± 0.2 g·mL^-1^ for PMMA particles, which perfectly agrees with the nominal values.

### Cell mass density measurements

**Figure 5:**
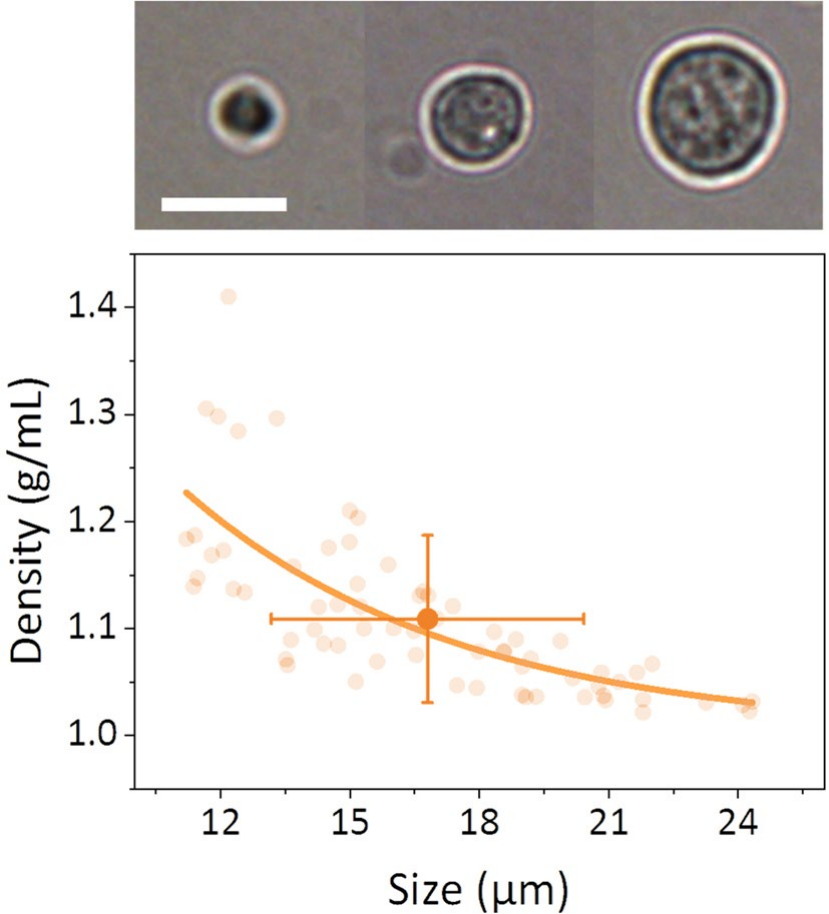
Cell mass density experiments. Up. Optical micrograph of individual MCF-7 cells of different sizes. The scale bar is 25 μm. Down. Experimental measurements of the mass density of individual MCF-7 human epithelial breast cells (orange symbols). The solid line represents a fitting of the experimental values, showing the size dependency of the measured density.

Mass density has been demonstrated as an interesting parameter for cell´s life-cycle characterization in previous works^26,27^ due to the relation of cell density with biological activity^28^. It is known that the cell density fluctuates during the cell cycle^35^. This is particularly important in human cells, where the density remains constant through the whole life-cycle except during mitosis^29,30^, when cells experience a rapid cell volume increase with the corresponding decrease in its mass density^36^. Therefore, there is a size dependency of the mass density, the larger cells showing a lower density. We have measured the density of MCF-7 (ATCC, USA) human breast adenocarcinoma cells (mean value of 1.11 ± 0.08 g·mL^-1^), Fig. 5, as a proof of principle of the capability of this mechano-optical technique to characterize cell density, together cell mass and cell reflectivity with high throughput. As seen from the experimental measurements, we observe a dependency of the density with cell size, which agrees with the results obtained in previous works about the variations observed during the cell life cycle^35,36^. This result proves the capability of the technique and opens the door for individual cell cycle studies by the multiparametric characterization proposed here.

## Discussion

In this work we have proposed a multiparametric particle spectroscopy method using suspended microcapillary resonators just by tracking the first flexural mode. We have theoretically and experimentally demonstrated that, despite being introduced with a random spatial distribution in the capillary device, particles will be eventually ordered in the sensing region as they will precipitate on the capillary wall as a consequence of lifting forces appearing. Additionally, drag and drive forces will determine the velocity of the particle depending on the pressure difference and the particle diameter, allowing to univocally relate particle velocity and diameter. Finally, we have tested these principles by measuring mixtures of particles of different diameters. Moreover, the device and the readout system employed in this work allows the optical probing of the flowing particles, which finally provides a triple parameter particle sensing. Simultaneous measurement of the buoyant mass through the frequency tracking and the particle size by means of its velocity and optical signal may be used to obtain information about the density of the flowing particles with high throughput, of up to 300 cells/min. We measured density variations of MCF-7 human breast adenocarcinoma cells, opening the door for individual cell cycle studies.

## Methods

### Cell culture

The cell lines MCF-7 from ATCC (USA) were grown in a mixture of DMEM (Dulbecco’s modified Eagle’s medium) (Life Technologies Corporation, USA), 10% FBS, 500 U/mL penicillin and 0.1 mg/mL streptomycin. Prior the measurements, the cells were trypsinized and resuspended, after soft centrifugation, in a volume of PBS with a 5% of DMSO, see reference 25 for further details.
